# Digital integrated systems in primary health care: advancing universal health coverage in low- and middle-income countries

**DOI:** 10.1016/j.ebiom.2026.106169

**Published:** 2026-02-11

**Authors:** Hadis Ashrafizadeh, Maryam Rassouli

**Affiliations:** a School of Nursing, Student Research Committee, Dezful University of Medical Sciences, Dezful, Iran; b School of Nursing, College of Health Sciences, University of Nizwa, Nizwa, Sultanate of Oman; c Cancer Research Center, Shahid Beheshti University of Medical Sciences, Tehran, Iran

Progress toward Universal Health Coverage (UHC) constitutes one of the foremost global health priorities, with all United Nations Member States having committed to its realisation by 2030.^1^ Achieving this objective necessitates rigorous attention to existing infrastructures and the implementation of strategies that effectively utilise current capacities to ensure the delivery of services with maximal accessibility and minimal financial hardship. Primary Health Care (PHC), represents a critical platform in many countries, offering a comprehensive framework for the provision of care grounded in an equitable, and cost-effective model.^2^

In many countries including Iran, efforts to advance UHC have a long history. For nearly five decades, PHC has been recognised as the cornerstone of the national health system and, through an extensive health care network, delivers health services under the supervision of medical universities across the country.^3^

Prior to the COVID-19 pandemic, this system played a substantial role in improving health indicators, including reductions in maternal and child mortality. Nevertheless, the pandemic exposed that the system faces considerable limitations including underdeveloped information systems and inadequate digital integration, emphasising the urgent need for a comprehensive revision.^4^

Redesigning this system must manage ongoing trends, including the rise in burden of non-communicable diseases, which represent the leading cause of mortality in the country. Accordingly, the advancement of digital health transformation initiatives and the adoption of technologies such as telemedicine are essential components in establishing an efficient PHC system.^5^

Findings from a recent study conducted in Rwanda and published in the March, 2026, issue of *eBioMedicine*, offer a compelling example of innovation within the PHC context.^6^ This experience illustrates that digital technologies can play a pivotal role in enhancing access, promoting equity, and improving the cost-effectiveness of health service delivery. In Rwanda, communicable diseases such as malaria, tuberculosis, pneumonia, and diarrhoea have long been central public health priorities and continue to account for a substantial share of the national disease burden. The integration of screening processes for these conditions into a unified digital system (d-IDS) not only facilitated Health Care Workers' efforts in patient identification but also promoted more equitable access to services through the existing community electronic medical record system (cEMR). Rwanda's experience provides a clear demonstration of how integrating digital screening systems at the PHC level can reduce unnecessary visits, increase the volume of screened cases, and ensure effective management at the primary point of care.

In Iran, digital health development has largely been pursued through the Electronic Health Record, yet the main challenge remains linking these systems to PHC programs. In the current system, the first level of health services has its own dedicated digital platform. However, the entry point for care is the health centres themselves, limiting the ability to track and comprehensively manage patients after hospital discharge.^7^ A clear example of this challenge is the IraPEN program, introduced in Iran as a localised version of the WHO Package of Essential Non-Communicable Disease Interventions.^8^ The program sought to strengthen national capacity to scale up care for a few non communicable illnesses like cardiovascular disease at the PHC level. In 2016, the Ministry of Health initiated its pilot phase in four cities.^8^ However, the absence of integrated digital infrastructures limited the program's full implementation.

A review of Iran's experience shows that even scientifically sound program designs require digital tools, and integrating the EHR with PHC programs and bridging existing gaps between service levels is a fundamental prerequisite for embedding IraPEN services into PHC, so that it may achieve the level of effectiveness observed in Rwanda's digital integration of communicable disease screening at the PHC level.^9^

Despite the substantial advantages associated with integrated digital systems within PHC, limited resources countries persistently encounter significant obstacles. These include unstable internet connectivity resulting from limited technological infrastructure in rural and underserved regions; constrained implementation capacity driven by financial limitations and shortages in the human resources required for system development, maintenance, and health worker training; and cultural as well as organisational resistance to technological change.^9^

In countries such as Rwanda, governmental leadership, stable digital infrastructures, and specialised implementation teams have played pivotal roles in advancing digital transformation. By establishing sustainable financing mechanisms, investing in the training and capacity-building of the health workforce, and developing digital innovation hubs, these countries have effectively laid the groundwork for systemic transformation. Based on such experiences, successful digital transformation necessitates strong governmental leadership and clearly articulated national policies.^10^

Evidence from comparative studies and successful experiences can be shared through regional and international networks, enabling countries to adapt and localise digital health models in alignment with their contexts. It is crucial to underscore that digital health within PHC should be regarded as a policy imperative, rather than merely a technological option, within both national and regional health agendas.

## Contributors

Both authors contributed to conceptualisation, writing, reviewing, editing, and have read and approved the published version of the commentary.

## Declaration of interests

The authors have no conflicts of interest to disclose.
